# Biomechanical Analysis of the Cross, Hook, and Uppercut in Junior vs. Elite Boxers: Implications for Training and Talent Identification

**DOI:** 10.3389/fspor.2020.598861

**Published:** 2020-11-26

**Authors:** Daniel Dinu, Julien Louis

**Affiliations:** ^1^French National Institute of Sport, Expertise and Performance, Sport, Expertise and Performance Lab, Paris, France; ^2^Research Institute for Sport and Exercise Sciences, Liverpool John Moores University, Liverpool, United Kingdom

**Keywords:** boxing, punch, inertial measurement unit, performance, combat

## Abstract

Punching in boxing requires a combination of force and velocity of the acting arm, originating from an optimal synchronization of the different body segments. However, it is not well-understood what kinematic parameters of the punching execution influence boxing performance the most. This study aimed to investigate the differences in punching execution between 15 potential Olympic medalist boxers (Elite group) and 8 younger well trained boxers (Junior group). Each athlete was equipped with an instrumented suit composed of 17 inertial measurement units (IMU) and were asked to perform several series of 3 standardized punch types (cross, hook, and uppercut) with maximal force. Linear velocity, stability, and punch forces were computed from the different sensors. Our findings show that Elite boxers systematically produced more force and at a higher velocity for the three punch types compared to Juniors. Further analysis revealed differences in joint contributions between Elite and Juniors, Juniors presenting a higher contribution of the shoulder for the three punch types. Finally ground reaction force imbalance between the front and rear foot was revealed in the cross only, in all boxers (60.6 ± 24.9 vs. 39.4 ± 24.9% and 54.1 ± 7.1 vs. 45.9 ± 7.1%, *p* ≤ 0.05, for the front vs. rear foot in Elite and Juniors, respectively) but not different between groups. These results have important implications for practitioners involved in the talent identification process, longitudinal follow-up, and training of boxers.

## Introduction

Boxing is a physically demanding combat sport. Boxers rely on a combination of strength, coordination, velocity, and stamina to succeed in impacting the opponent while evading adversary punches (Whiting et al., 1988; Mack et al., 2010). A successful performance requires the ability to deliver precise punches above the belt, to the head or the torso without being punched back. In amateur boxing, such seen during the Olympic Games, boxers aim to score by striking the opponents during rounds of 3 min. As varying degrees of force and velocity are required in boxing, athletes, throw punches with their rear or front hand (Kimm and Thiel, 2015). The rear hand (the furthest from the target) usually provides more punching force while maximal velocity can be achieved with the front hand (the closest to the target) (Dyson et al., 2008). The defensive boxer is allowed to dodge punches with hand, trunk as well as feet actions. There are three main attacking techniques: the cross, hook, and uppercut. The cross implies a forward translation of the body whereas the two other punches involve an overall rotation of the body. Previous studies have reported an activity rate of ~1.55 actions/s, consisting of ~21 punches, ~3.6 defensive movements, and ~56 vertical hip movements per-minute over three subsequent rounds lasting ~184 s for male elite boxers (Davis et al., 2015, 2016).

During the round, boxers aim to knock their opponent out, touching the optimal target zone in order to win the fight. Because knockout is a constant goal during a match, boxers must increase punch impact and, therefore, knockout power (Cheraghi et al., 2014; Chaabene et al., 2015; Loturco et al., 2016). Punching force and velocity are, therefore, major determinants of performance in boxing, with higher maximal values generally reported for higher level boxers (Smith et al., 2000). Unlike professionals, amateur boxers tend to favor quick strikes over heavy blows, potentially to compensate for lower muscle force (Cheraghi et al., 2014). Therefore, they need to develop maximal velocity at the end of the distal segment of the kinematic chain. In this aspect, boxing generates the same type of segment interactions as sports involving throwing and kicking such as discus throw, softball, tennis, or baseball (Elliott et al., 2003; Rojas et al., 2009; Oliver and Keeley, 2010; Dinu et al., 2019). Achieving high velocity, and force, at the end of the distal segment is usually a result of a proximal-to-distal sequencing motion as the summation of velocity principle states (Cabral et al., 2010; Zhang et al., 2011; Cheraghi et al., 2014). However, computation of sequencing motion remains a challenge (Marsan et al., 2019). The synchronization of the body segments' motion can highlight the differences in skills between athletes (Putnam, 1993). Hence, understanding these biomechanical differences can provide valuable insight for lower level athletes and coaching staff desiring to refine their training practices.

The aim of our study was to investigate the differences in punching execution between Elite and Junior amateur boxers during the cross, hook, and uppercut. Based on previous research, we hypothesized that Elite boxers would display higher punching forces and velocities, accompanied with specific body segment contributions different from Junior athletes. The results of this study have important implications for the athletes and coaches for development purposes.

## Materials and Methods

### Participants

Two groups of male amateur boxers volunteered to participate in this study: 15 elite potential Olympic medalist boxers (Elite) (mean age: 21.1 ± 3.0 years; height: 1.79 ± 0.09 m; body mass: 73.6 ± 17.9 kg) from the National boxing academy and 8 junior boxers (Junior) (mean age: 16.1 ± 0.7 years; height: 1.75 ± 0.05 m; body mass: 61.0 ± 9.3 kg) from a regional boxing academy. All the participants from the Elite group had been competing at the highest International level in their category for a minimum of four consecutive years and were part of the National team preparing for the next Olympic games. The athletes of the Elite group trained daily at the National boxing academy. On the contrary, participants of the Junior group had a lower level experience and trained less often in their regional boxing clubs. All the participants were injury free at the time of the data acquisition. This study was approved by the National Boxing Federation and the local ethics committee (Paris, IDF, France). All the experimentations were carried out in accordance with the Declaration of Helsinki. Participants were fully informed of the objectives and risks of the study and their parent or legal guardian signed an informed consent form before the study began.

### Protocol

Prior to testing sessions, a standardized warm-up was organized under the supervision of the coach. The participants were asked to perform 3 punches using standardized techniques (cross, hook, and uppercut) with, at first, their front hand, then, their rear hand, and finally, a combo: front hand immediately followed by rear hand. A series of 3 punches was executed for each technique. The instructions were to complete a precise motion in the direction of the punching bag with maximal possible strength, as classically performed during their habitual training sessions. Participants wore an MVN Biomech Link suit (Xsens Technologies BV, Enschede, The Netherlands) collecting live kinematic data during the entire movement (Louis et al., 2018; Figueiredo et al., 2020). This suit was composed of 17 miniature inertial measurement units (IMU) strapped onto the body. Each IMU contained a 3D gyroscope, a 3D accelerometer and a 3D magnetometer in an 18 g box (about the size of half a matchbox 3.5 × 2.5 × 0.8 cm). Each IMU captured the 6 degrees of freedom of the body segment to which it was fixed, in real time at a sampling frequency of 240 Hz.

### Data Processing

Based on the linear velocity and acceleration of each segment computed from the IMU, a customized MatLab™ program (7.10.0, R2010a, Natick, USA) calculated the estimate of the ground reaction force distribution and the punch force at impact. All biomechanical analyses were performed according to the De Leva anthropometrical model (De Leva, 1996).

This study concentrated on three mechanical parameters: the linear velocity at impact accessed *via* the hand's IMU, the distribution of ground reaction forces at impact. The determination of ground reaction forces and punching force is detailed below.

#### Distribution of Ground Reaction Forces Computation

Vertical ground reaction forces (GRF) of the left and right foot were estimated *via* the projection of the center of mass, as proposed in Equation (1).

(1) $$\begin{array}{l} \vec{GRF}=\text{m}\left(\;\vec{a_{CMz}}-\vec{g\;}\right) \end{array}$$

In this equation, based on Newton's second law, $\vec{GRF}$ corresponds to the total ground reaction forces, m is the mass of the athlete, $\vec{a_{CMz}}\;$is the vertical component of the center of mass acceleration obtained by the IMU, $\vec{g\;}$ corresponds to the gravitational acceleration.

In order to study the leg which was the most involved during the motion and to measure the athlete's balance during the motion, the GRF distribution between the lead and rear leg was computed based on a proportional distribution of the toes. First, the center of mass was calculated from the sum of the center of mass of each body segment and then projected onto the ground. Then the distance between the projected center of mass and the toes (respectively, *d*_*CM*−*L*_ and *d*_*CM*−*R*_ for the left and the right foot) was measured with the kinematic data acquired by the IMU. *d*_*Total*_ corresponded to the distance between both feet (2). GRF distribution on the lead foot (${\left\|\vec{GRF}\right\|}_{L}$) and on the rear foot (${\left\|\vec{GRF}\right\|}_{R}$) was computed following the Equations (3) and (4) and was presented as a percentage of $\left\|\vec{GRF}\right\|$. GRF distribution of the lead foot was used to estimate the boxer's balance at impact, with the athlete being the most stable when the GRF distribution of the front leg was close to 50%.

(2) $$\begin{array}{l} d_{Total}=\;d_{CM-L}+\;d_{CM-R} \end{array}$$

(3) $$\begin{array}{l} {\left\|\vec{GRF}\right\|}_{L}=\;\frac{d_{CM-L}\;\times \|\vec{GRF}\|}{d_{Total}} \end{array}$$

(4) $$\begin{array}{l} {\left\|\vec{GRF}\right\|}_{R}=\;\frac{d_{CM-R}\;\times \|\vec{GRF}\|}{d_{Total}} \end{array}$$

#### Punching Force Estimation

When computing the punching force, GRF was assumed constant between the moment preceding the impact and the impact. Thus, following the hypothesis that the lateral ground contact forces are negligible, it is possible to write Newton's second law prior to impact and at impact (Murata, 2001). The punching force at impact $\vec{F}$ can be singled out and calculated: $\|\vec{F}\|+\text{ ma}=\;\frac{\text{ΔP}}{\text{Δt}}$ (5) where $\left\|\vec{F}\right\|$ is equivalent to the magnitude of the impact force of the punching bag on the boxer's hand, ${\left\|\vec{GRF}\right\|}_{R}$ and ${\left\|\vec{GRF}\right\|}_{L}$ match the ground reaction forces, m is the boxer's weight, *P* corresponds to the linear momentum of the boxer, t is the time frame, $\vec{g}$ is the gravitational acceleration and $\vec{a}$ is the acceleration of the center of mass of the boxer at the moment prior to impact.

#### Contribution of Body Segment Calculation

The contribution of body segments was computed by the analytic calculation of the velocity of the segment of interest. A kinematic chain was built from the reference point, in this case it was the pelvis, to the segment of interest in the form of (6) (Zhang et al., 2011). The linear velocity of the kinematic chain was based on the linear velocity of the reference point. The segment angular velocity describes the other segments between the reference point and the segment of interest.

(6) $$\begin{array}{l} {\vec{V}}_{seg\;x}={\vec{V}}_{pelvis}+{\vec{\omega }}_{pelvis}\times {\vec{L}}_{pelvis}+{\vec{\omega }}_{seg\;1}\times {\vec{L}}_{seg\;1}+\ldots \\ \;+{\vec{\omega }}_{seg\;x-1}\times {\vec{L}}_{seg\;x-1} \end{array}$$

$\overset{⇀}{V}$ represents the linear velocities, $\overset{⇀}{\omega }$ represents the 3D segment angular velocities. The length $\overset{⇀}{L}$ corresponds to the 3D vector between ${\overset{⇀}{V}}_{seg\;x}$ and the proximal joint of the chain.

The kinematic chain involved in punching consists of the pelvis, the trunk, the shoulder, the elbow, and the wrist. The contribution of each body segment was found by projecting the velocity vector of the segment on the velocity vector of the wrist. For example, the projected velocity of the upper arm has been calculated in 7 and 8.

(7) $$\begin{array}{l} {\overset{⇀}{V}}_{upper\;arm\;proj.}=\frac{{\overset{⇀}{V}}_{upper\;arm}{•\overset{⇀}{V}}_{hand}}{{\left\|{\overset{⇀}{V}}_{hand}\right\|}^{2}}\times {\overset{⇀}{V}}_{hand} \end{array}$$

(8) $$\begin{array}{l} {\overset{⇀}{V}}_{upper\;arm}=\;{\overset{⇀}{\omega }}_{upper\;arm}\times {\overset{⇀}{L}}_{upper\;arm} \end{array}$$

${\overset{⇀}{V}}_{upper\;arm\;proj.}$ is the projected velocity vector of the upper arm. ${\overset{⇀}{V}}_{upper\;arm}$ is the velocity vector of the upper arm and ${\overset{⇀}{V}}_{hand}$ that of the hand. ${\overset{⇀}{\omega }}_{upper\;arm}$ is the 3D segment angular velocity vector of the shoulder, and ${\overset{⇀}{L}}_{upper\;arm}$ is the 3D vector between the velocity vector of the upper arm and the wrist.

### Statistical Analysis

As the participants had three attempts for each punch type, only rear hand data for the best punch (i.e., allowing the highest force production) were included in the analysis (Smith, 2006). Prior to data analysis, a Shapiro-Wilk test was used to assess normality of distribution, with punching force (relative to body mass) for the cross, punching speed for the hook and uppercut, segment contributions, joint angles, and GRF for all punch types normally distributed; and punching force (relative to body mass) for the hook and uppercut, punching force (absolute value) for all punch types, and punching speed for the cross not normally distributed. For each punch type (Cross, Hook, and Uppercut), between-group (Elite vs. Junior) differences in punching force, punching velocity, joint angles, GRF, and the position of CM, were analyzed using an independent *t*-test, or a non-parametric Mann-Whitney *U*-test, when data were normally or not normally distributed, respectively. Segment contributions to the different punch types were evaluated between boxing groups using a repeated analysis of variance and adjusted using the Bonferroni *post-hoc* test between groups and body segments if a significant interaction was indicated for the different segments. Cohens *d* [95% CI] was calculated for effect size where normality was met and was subsequently assessed using the following thresholds: <0.2 = trivial effect; 0.2–0.6 = small effect; >0.6–1.2 = moderate effects; >1.2–2.0 = large effect; >2.0–4.0 = very large effect and >4.0 = extremely large effect (Hopkins et al., 2009). In circumstances where normality was not displayed, the *r* (uses *z*-score from Mann-Whitney *U*-test) was used for effect size (Fritz et al., 2012). The *r* was interpreted from Cohen's criteria where: 0.1 = small effect; 0.3 = moderate effect; and 0.5 = large effect. Pearson's correlation coefficients were also calculated to determine the relationship between punching force and velocity. For all statistical analyses, a *p*-value of 0.05 was considered to indicate significance. All data are presented as means ± standard deviations (SD), unless otherwise indicated. All statistical analyses were performed using Statistical Package for the Social Sciences (SPSS, Version 25, IBM, New-York, USA).

## Results

### Punching Force and Velocity

**Figure 1:**
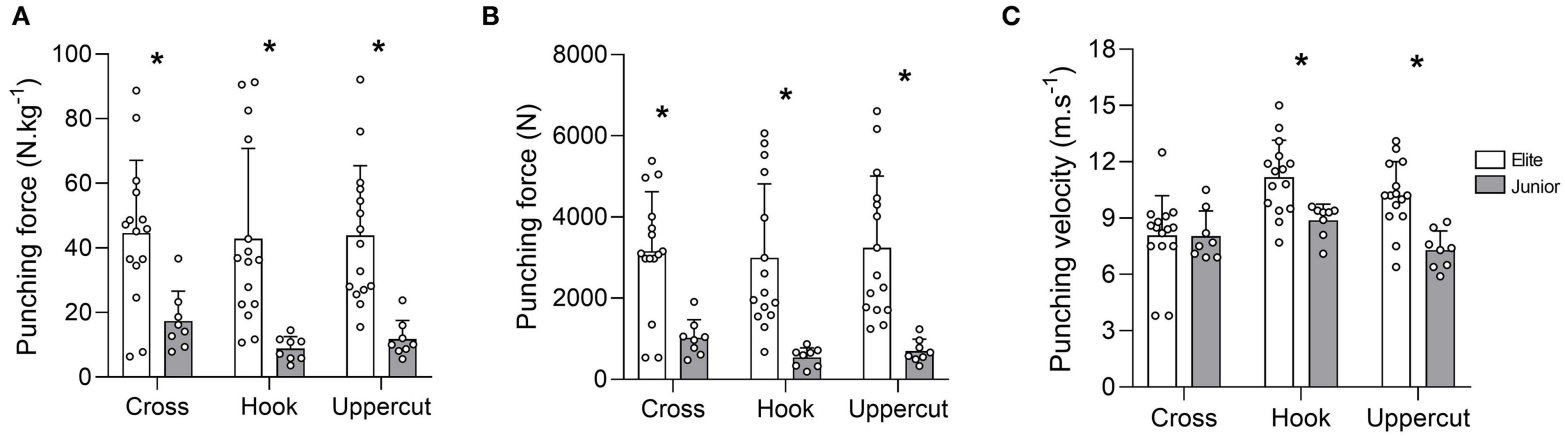
**(A)** punching force (N.kg^−1^), **(B)** punching force (N), **(C)** punching velocity (m.s^−1^) for the different punch types for the two groups of boxers (in **A–C**; *n* = 8 and 15 in Junior and Elite groups). Bars represent mean values, error bars represent SD values, and white circles represent individual data points. * denotes a significant difference between groups (*p* ≤ 0.05).

For all punch types, maximal force production was higher (*p* < 0.01) in Elite compared to Junior boxers (Figures 1A,B). The mean maximal force produced by Elite vs. Junior boxers was 3,158 ± 1,467 vs. 1,021 ± 449 N, *r* = 0.6 [−0.2; 1.5], 2,999 ± 1,818 vs. 544 ± 235 N, *r* = 0.8 [−0.1; 1.6], and 3,242 ± 1,767 vs. 700 ± 287 N, *r* = 0.8 [−0.07–1.7] for the cross, hook, and uppercut, respectively. As displayed in Figure 1A, the results followed the same pattern between Elite and Juniors when force production was considered relative to individual body mass (in N.kg^−1^). The punching velocity was also higher (*p* < 0.01) in Elite compared to Juniors for the hook and uppercut only (Figure 1C). The mean maximal punching velocity in Elite vs. Junior boxers was 8.1 ± 2.1 vs. 8.1 ± 1.3 m.s^−1^, *r* = 0.1 [−0.7–1.02], 11.2 ± 2.0 vs. 8.9 ± 0.9 m.s^−1^, *d* = 1.1 [0.2–2.1], and 10.2 ± 1.8 vs. 7.3 ± 1.0 m.s^−1^, *d* = 1.4 [0.4–2.3] for the cross, hook, and uppercut, respectively.

**Figure 2:**
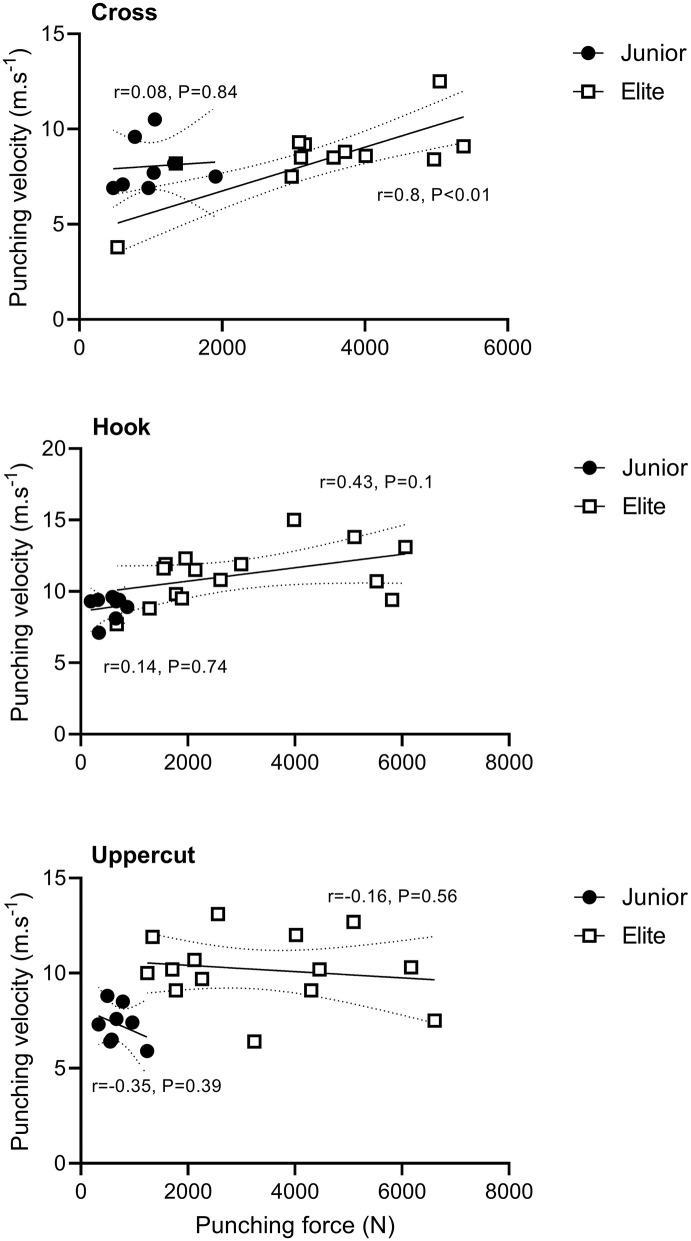
Relationship between punching velocity and punching force for the different punch types for the two groups of boxers (in all panels, *n* = 8 and 15 in Junior and Elite groups). Straight lines represent the best fit and dotted lines represent the 95% CI. Points represent individual values for each boxer in the Elite (white squares) and Junior (black circles) groups. In each panel, r is the Pearson correlation coefficient.

Punching velocity was positively correlated with punching force (*r* = 0.8 [0.5–0.9], *p* < 0.01) for the cross only and in Elite boxers only (Figure 2). However, there was no significant correlation between punching force for all punch types and body weight in both groups (*p* > 0.05 for all analyses).

### Technical Aspects of the Punch

**Figure 3:**
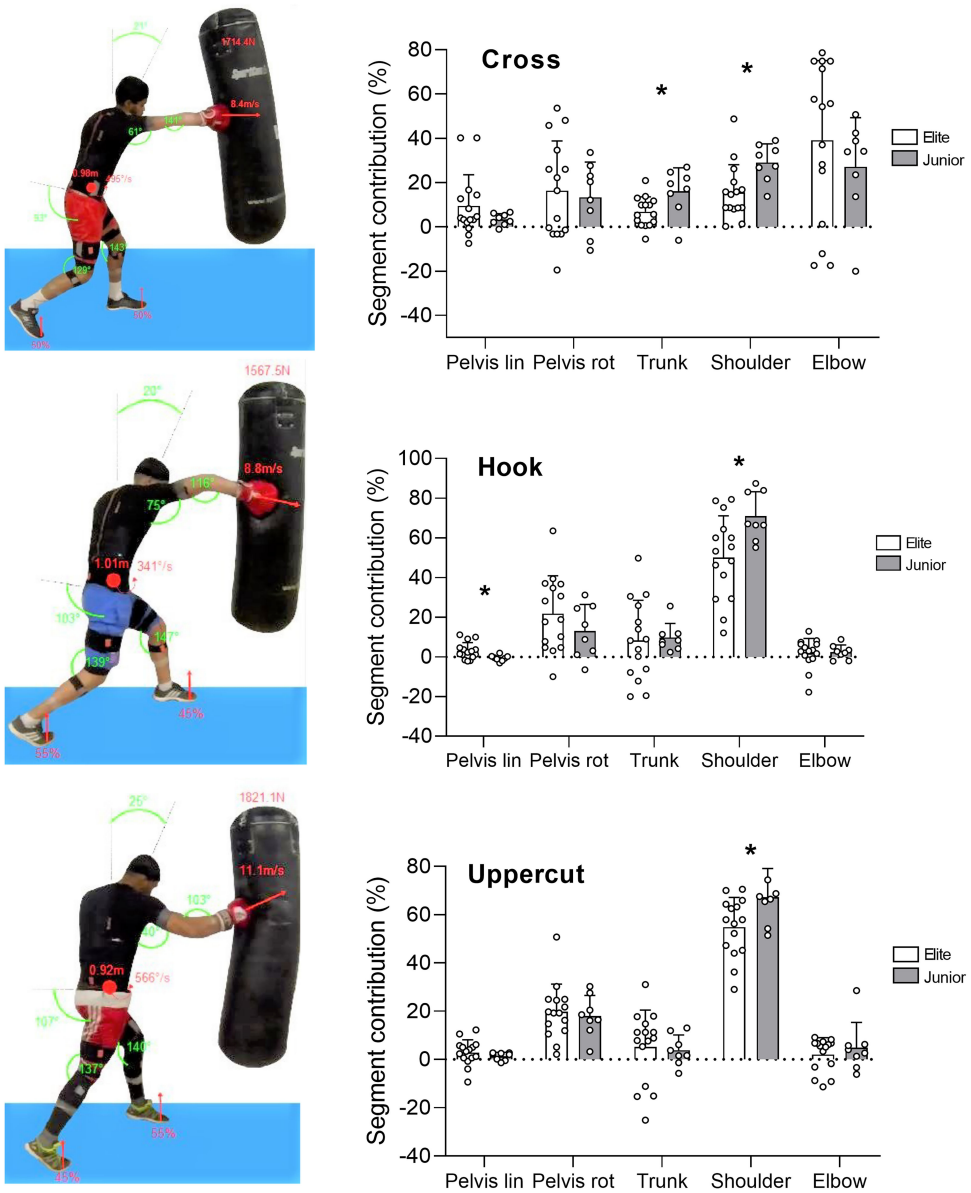
Examples of 3D avatars reconstructed from live kinematic data and corresponding segments' translation and rotation contributions (%) for the three punch types for the two groups of boxers (in all panels, *n* = 8 and 15 in Junior and Elite groups). Avatars are visual example only and are not representative of the participants of the study. Segments' contributions for trunk, shoulder, and elbow are a combined translation and rotation. Bars represent mean values, error bars represent SD values, and white circles represent individual data points. * denotes a significant difference between groups (*p* ≤ 0.05).

The body segments' contributions showed different patterns between punches and between groups (Figure 3). In both groups, the elbow contributed the most to the punch during the cross (39.2 ± 35.9% and 27.1 ± 22.2% for Elite and Junior, respectively), whereas it was the shoulder that contributed the most to the execution of the hook and uppercut. Besides, the shoulder contribution was systematically higher in Junior compared to Elite for the cross (29.1 ± 8.4 vs. 15.6 ± 12.5%, *p* = 0.01, *d* = 1.04 [0.1–1.9]), hook (71.0 ± 12.3 vs. 50.1 ± 21.0%, *p* = 0.01, *d* = 1.0 [0.1–1.9]) and uppercut (67.3 ± 11.9 vs. 54.8 ± 12.3%, *p* = 0.02, *d* = 0.9 [0.03–1.8]). The trunk contribution was also higher in Junior compared to Elite only for the cross (16.0 ± 10.6 vs. 6.7 ± 6.8%, *p* = 0.01, *d* = 1.0 [0.09–1.9]). The pelvis contribution in the linear plane was higher (*p* = 0.02, *d* = 0.9 [0.04–1.8]) in Elite (3.04 ± 4.2%) compared to Junior (−0.6 ± 1.5%) during the hook only.

The 3D kinematic analysis also showed no difference in body positioning between groups for the three punch types, except for the neck angle which was lower (*p* = 0.01, *d* = 1.0 [0.1–1.9]) in Junior (19.8 ± 4.9°) compared to Elite (24.0 ± 3.0°) during the cross (Table 1). GRF showed an imbalance between the lead and rear foot only during the cross (*p* ≤ 0.05) and in similar proportions between groups. The mean GRF contributions in Elite boxers was 60.6 ± 24.9 vs. 39.4 ± 24.9% (*d* = 0.8 [0.09–1.7]) for the lead vs. rear foot, respectively, and 54.1 ± 7.1 vs. 45.9 ± 7.1% (*d* = 1.02 [0.1–1.7]) for the lead vs. rear foot, respectively.

**Table 1:** Joint angles for the elbow, neck, and knee (°), position of the center of mass (CM, m) and contributions of ground reaction (%) for the lead and rear foot, for the two groups of boxers (*n* = 8 and 15 in Junior and Elite groups).

		**Cross**	**Hook**	**Uppercut**
Elbow (°)	Elite	112.9 ± 9.2	116.9 ± 22.6	100.1 ± 12.6
	Junior	120.0 ± 12.4	113 ± 18.1	94.5 ± 16.6
Neck (°)	Elite	23.1 ± 2.5^*^	23.6 ± 4.9	20.9 ± 3.4
	Junior	19.8 ± 4.9	21.9 ± 4.1	18.0 ± 4.6
Knee (°)	Elite	142.8 ± 16.1	143.6 ± 9.5	145.7 ± 12.3
	Junior	146.6 ± 6.2	144 ± 7.3	141.0 ± 8.0
CM height (m)	Elite	0.9 ± 0.1	0.9 ± 0.1	0.9 ± 0.1
	Junior	0.9 ± 0.05	0.9 ± 0.0	0.9 ± 0.1
GR lead foot (%)	Elite	60.6 ± 24.9^#^	58.9 ± 25.5	49.9 ± 14.6
	Junior	54.1 ± 7.05^#^	46.1 ± 11.2	53.0 ± 9.81
GR rear foot (%)	Elite	39.4 ± 24.9	41.1 ± 25.5	50.1 ± 14.6
	Junior	45.9 ± 7.05	53.9 ± 11.2	479 ± 9.81

* *denotes a significant difference between groups (p ≤ 0.05)*,

# *denotes a significant difference between lead and rear foot (p ≤ 0.05)*.

## Discussion

Performance in boxing requires a combination of force and velocity of the acting arm, originating from an optimal synchronization of the different body segments. We examined the biomechanical patterns and resulting punching forces and velocities produced by Elite vs. Junior boxers for three punch types. Our main findings reveal differences in punching force, punching velocity, and body segment contributions between groups, thus better informing on the conditions required to perform in boxing. Interestingly, ground reaction forces were different between the lead and rear foot, thus creating an imbalance during the cross in all boxers, with no significant differences between groups. Body positioning in space was not different between groups as inferred through the analysis of elbow, neck, and knee angles during the punch.

From the 3D kinematic analysis, we identified no marked differences in body positioning between groups during the three punch types at impact. Joint angles and the position of the center of mass were not different between boxers (except for the neck angle during the cross). In contrast, differences in motion patterns between the hook, uppercut and cross were recorded, across the two groups of boxers. We found that in both groups, at impact time, the elbow was the upper-body segment that contributed the most to the execution of the cross, while it was the shoulder during the hook and uppercut. The cross requires a straight trajectory with the elbow acting like a piston (flexion-extension) in a throwing movement performed in the sagittal plane and with very little rotation. The cross is considered as a short movement requiring the opening of the elbow to reach the target. In contrast, the hook and uppercut are longer and more complex. They require a circular trajectory in the sagittal plane with the shoulder predominantly mobilized to initiate a simultaneous rotation and translation of the arm (Whiting et al., 1988). These differences in segment contributions observed in both groups between the cross and hook/uppercut show that the different punching techniques require very distinct biomechanical adjustments. Interestingly, moderate (effect size*, d* > 0.6–1.2) differences in segment contributions were also observed between groups of boxers. Overall, some body segment contributions were systematically higher in Junior compared to Elite boxers, especially for the shoulder. As depicted in Figure 2, trunk and shoulder during the cross, pelvis, and shoulder during the hook, and the shoulder during the uppercut, contributed the most to the punch at impact time in Junior compared to Elite boxers. Given that Junior systematically produced less force than Elite boxers, body segment contribution data reveal that they were less effective than Elite boxers. It can be hypothesized that these different biomechanical contributions in the execution of the movement be explained by differences in level of expertise and technique. In other throwing sports such as discus throwing, a higher technique variability was reported in lower level athletes (Dai et al., 2013). Although we did not measure any indices of variability of execution, we can reasonably hypothesize that intra-individual variability in punching technique could have been higher in Junior boxers. A higher activation of muscles not directly involved in movement production was also reported in amateur compared to professional baseball pitchers, leading to a less efficient pitch (Gowan et al., 1987). We also think that junior boxers engaged more some segments in the punch in an attempt to compensate for their lack of punching force and velocity compared to elite boxers. Finally, between-groups differences in lower-body force production capacity might also explain the different upper-body segment contributions recorded in our study.

Leg drive and foot positioning are very important in boxing to facilitate energy transfer from the lower body to upper limbs, thus facilitating force production. The movement initiates at the feet, through a lead foot lift and rear leg drive. Although only few studies have focused on this parameter, it is generally accepted that the greater the legs' contribution, the greater the punching force and this pattern is more prominent in experienced boxers (Filimonov et al., 1985). In general, experienced boxers increase this forward movement (i.e., lifting the lead foot forward and increasing lead foot pressure on the ground) thanks to a concomitant rear leg extension (Filimonov et al., 1985). A similar leg drive was also reported in baseball pitching with the fastest pitches associated with the highest front leg contributions (Macwilliams et al., 1998). In our study, we analyzed leg contributions to the punch through the distribution of GRF between the lead and rear feet. Our data show rather similar patterns between groups with the lead foot systematically showing the highest values compared to the rear foot especially during the cross (moderate effect sizes in both groups). This pattern tended to be accentuated in Elite compared to Juniors during the cross and hook, but this was not significant (Table 1). The balance between GRF for the lead and rear foot as well as leg drive is important in boxing in order to facilitate energy transfer from the lower body to upper limbs, thus facilitating force production. Given the present study is one of the first to differentiate the cross, from the hook and uppercut in its analyses, specific comparisons of each punch type with the literature are not possible. Nevertheless, in our study, it is noteworthy that a balanced GRF distribution between the lead and rear foot (with a 50/50% distribution colloquially stated as best technique) was considered by coaches as an indicator of good punching execution and performance. Although this might be the case in a boxing match for a better positioning on the ring or to maintain a good balance in front of an opponent, this opinion was not confirmed by our data and previous data collected in laboratory conditions. In novice amateur boxers, Stanley et al. (2018), reported that the lead or rear leg may present a higher or lower GRF depending on the punch type and technique of execution (i.e., rear or lead hand). As recorded in our study, and based on other throwing activities such as the shot put, discus throw, javelin, and baseball, it seems that a stable lead leg (presenting the highest GRF) be necessary to produce force proximally to the end of the kinematic chain (i.e., the most distal segment which is the fist) (Whiting et al., 1991; Bartonietz, 1994; Macwilliams et al., 1998; Dinu et al., 2019). From a practical perspective, these data confirm the importance of lower limb force development in boxers. Giovani and Nicolaidis (2012) already showed that boxers with higher maximal power in the lower limbs also presented higher maximal power in the upper limbs. Accordingly, training programs should aim to develop both the upper and lower limb muscle force of boxers. Loturco et al. (2019) recently confirmed the beneficial effects of a short-term power-oriented training program of lower limbs on performance of elite boxers. In the latter study, only three resistance training sessions including bench press, half-squat and jump squat exercises increased punching (jab and cross) impact forces by ~8%, and with an effective transference of ~0.80.

In both groups, force production was relatively similar between punch types, while the punching velocity tended to be higher during the hook, which can be explained by the swinging nature of the movement. Not surprisingly, force production was higher (large effects, *r* > 0.5) in Elite compared to Junior boxers for the three different punches regardless of the body mass of the athletes (Smith et al., 2000; Lenetsky et al., 2013). The force values recorded in the Elite group (≥3,000 N) were in accordance with values reported in the literature (3,427 ± 811 N) for Olympic boxers (Walilko et al., 2005). As knock out is an important factor of performance in match, the development of maximal force production must be prioritized in boxing training. Punching velocity is also very important in combination with a high force production in order to reach the highest possible punch power at impact. In our study, punching velocity was also higher in Elite compared to Junior boxers during the hook (moderate effect, *d* = 1.1) and uppercut (large effect, *d* = 1.4). Interestingly, in the two groups of boxers, neither body mass nor height were correlated with punching force or velocity. Accordingly, and contrarily to the results of Walilko et al. (2005), differences in punching force and velocity between groups could not be explained by differences in weight categories nor stature. Similarly, when we presented punching data according to body mass (in N.kg^−1^), punching force was still higher in Elite compared to Junior boxers. Our data also revealed a strong positive correlation (*r* = 0.8) between punching velocity and punching force for the cross in elite boxers only, reinforcing the fact that a high punching force requires a high speed of execution. In our study the mean punch velocity, including all punch types was 9.8 ± 2.3 and 8.1 ± 1.2 m.s^−1^ in Elite and Juniors, respectively, which is in line with values reported in the literature for boxing, karate and kung-fu (Wilk et al., 1983; Neto et al., 2007; Lenetsky et al., 2013). In addition, the higher punching force and velocity in Elite compared to Juniors might be explained by a greater contribution from the legs to the punch (Filimonov et al., 1985) and/or a higher force production capability of lower body segments (Loturco et al., 2016, 2019).

The data presented in this article have important practical implications for practitioners, in the detection of young talents, longitudinal follow-up and training of athletes. From a training standpoint, Junior boxers should take example from their elite counterparts to fill the gap in performance between them. It seems that training should mainly be directed toward the improvement of punching force and punching velocity. In our study, Juniors were able to produce only about one third of the force produced by Elite boxers, while the punching velocity was closer to that of Elite boxers but still significantly lower. Although technical aspects are important contributors to punching performance, it is important to consider maturation variables too. Indeed, Juniors presented a lower body mass compared to Elite boxers. Even though we did not measure it, it is likely that muscle mass was also lower in Juniors compared to Elite boxers, which could have explained the differences in force production and segment contribution during punches. Corroborating these hypotheses, in a recent study, Lopez-Laval et al. (2020) reported that the movement velocity recorded on a bench press exercise at 80% of one maximal repetition was correlated with the punching velocity of the rear arm during the punch in professional boxers. Even though the punch type was not specified in the latter study, these results suggest that a combination of punching force and velocity are crucial to perform optimally in boxing and it can be tested through gym-based exercises. In an earlier study, Loturco et al. (2016) also showed a strong correlation between strength/power of both upper and lower limb muscles recorded in various gym-based exercises and punching impact force in elite amateur boxers.

Although valuable biomechanical data were obtained during different punch types in Junior and Elite boxers, it is important to consider the limitations of the present results. Punching force was estimated through mathematical modeling and not directly measured at impact, which might have slightly over- or under-estimated force production values. All the biomechanical analyses were conducted at the moment of impact which was considered as the most representative of performance in boxing. Additional studies will be necessary to investigate all the kinematic events (from initiation of the movement to contact of the fist on the target) occurring during the punch in elite boxers. Another limitation to consider is that the technical level of our athletes was not assessed which could have also influenced the data recorded in both groups. In fact, the only inclusion criterion considered in the current study was the selection in the National team or Regional training clubs for Elite and Junior groups, respectively. Finally, the present study was conducted in a standardized environment where boxers had to punch a boxing bag, hence we can reasonably expect different biomechanical adjustments, punching force and velocities in live combat situations. Accordingly, future research should extend the analysis to boxing situations including opponents.

## Conclusion

This study examined the biomechanical differences between Elite and Junior boxers during three different punch types (cross, hook, and uppercut). Nanotechnology inertial measurement units were positioned directly onto body segments to provide a full decomposition of the biomechanical variables associated with the boxing tasks. Results indicated differences in punching force, punching velocity, and body segment contributions between the two groups. These findings allow to highlight the best punching techniques, thus providing valuable information for practitioners to refine their training practices, for the detection of young talents and longitudinal follow-up.

## Data Availability Statement

The raw data supporting the conclusions of this article will be made available by the authors, without undue reservation. Data will be made available upon request made to the corresponding author.

## Ethics Statement

The studies involving human participants were reviewed and approved by National Boxing Federation and the local ethics committee (Paris, IDF, France). Written informed consent to participate in this study was provided by the participants' legal guardian/next of kin.

## Author Contributions

DD and JL conceived and designed the study, performed data collection, processing, analysis, drafted the manuscript, and reviewed and accepted the final version of the manuscript. All authors contributed to the article and approved the submitted version.

## Conflict of Interest

The authors declare that the research was conducted in the absence of any commercial or financial relationships that could be construed as a potential conflict of interest.
